# Social theory and infant feeding

**DOI:** 10.1186/1746-4358-6-7

**Published:** 2011-06-15

**Authors:** Lisa H Amir

**Affiliations:** 1 Mother & Child Health Research, La Trobe University, Melbourne, Australia; 2 Centre for Women's Health, Gender and Society, University of Melbourne, Melbourne, Australia

## Abstract

Clinicians, public health advisors, nutritionists and others have been attempting to increase breastfeeding rates for the last few decades, with varying degrees of success. We need social science researchers to help us understand the role of infant feeding in the family. Some researchers in the area of food and nutrition have found Pierre Bourdieu's theoretical framework helpful. In this editorial, I introduce some of Bourdieu's ideas and suggest researchers interested in infant feeding should consider testing these theories.

## Editorial

Clinicians, public health advisors, nutritionists and others have been attempting to increase breastfeeding rates for the last few decades, with varying degrees of success [1]. However, health-related behaviours do not occur in isolation: by recognising the importance of social circumstances we can improve our understanding of infant feeding, thereby improving our ability to increase breastfeeding in our communities.

Social scientists can teach us about infant feeding behaviour. Biological anthropologists have compared humans with other primates to estimate the natural duration of breastfeeding in humans [2]. McKenna and colleagues have also used nonhuman primate data, cross-cultural studies and physiological studies to examine the natural ecology of mother-infant sleep [3]. A recent symposium on evolutionary anthropology has further explored this new area of research [4]. Other social scientists have contributed to our understanding of breastfeeding in the context of women's lives [5-9]. Our recent thematic series 'Infant feeding and HIV: lessons learnt and ways ahead' highlights the multiple challenges that HIV-infected women, infant feeding counsellors and health systems face in translating policy - in fact, changing policies - into practice [10]

I recently read the work of some researchers in the area of food and nutrition who have found Pierre Bourdieu's theoretical framework to be helpful. Warin and colleagues explain "To place food in social context resonates with Bourdieu's (1979/1984) study of food and social class in France. In *Distinction: a Social Critique of the Judgement and Taste*, Bourdieu argues that food and eating is much more than a process of bodily nourishment: it is an elaborate performance of gender, social class and identity" [11] (p. 98). Danielle Groleau and Charo Rodriguez have found some of Bourdieu's concepts helpful in interpreting their ethnographic interviews of disadvantaged French-Canadian mothers [12]. In this editorial, I aim to introduce Bourdieu's social theory and suggest other researchers interested in infant feeding consider testing its usefulness.

'Lay knowledge', that is the meanings and experiences influenced by the social circumstances in which people live, is considered a more useful concept than 'attitudes' or 'beliefs', as it acknowledges that individuals exist within a social environment [13]. The Adelaide Families and Food Study set out to contrast lay knowledge and eating habits of people from different social backgrounds [13]. The researchers recruited 20 families with at least one child less than 12 years of age from each of two areas: one area with low-income/subsidised housing/low tertiary education and one area with high-income/tertiary education common [13]. Low income families referred to more functional aspects of food (growth, stamina, vitality); they were "more likely to see food as a means of fuel and an immediate source of sustenance" [[13] p.294]. High income families referred to vitamins, fibre, risk of disease; they were "more inclined to express a scientific, more abstract, nutritionally informed understanding of food" [13] (p. 294).

Marshall and colleagues found some families assess feeding in terms of the baby's behaviour: a contented, thriving infant signifies 'good mothering' [14]. Lower income families want to ensure the baby is well fed, satisfied, settled, and gaining weight. A study of low-income Latino families in New York City described how one mother supplemented her children's formula bottles with cereal from infancy: "And like other mothers, Adela is clear about why she supplements her children's eating: food keeps her children happy" [15] (p. 2186).

Coveney's [13] findings in the Adelaide Families and Food Study align with Bourdieu's study of social class in France: "... whereas the working-classes are more attentive to the strength of the physical (male) body than its shape, and tend to go for products that are both cheap and nutritious, the professionals prefer products that are tasty, health-giving, light and not fattening" [16] (p. 593) citing [17]. Bourdieu's concept of *habitus *is his way of describing the embodiment of social structures and history in individuals; individuals perceive and act according to their social background [18]. "An individual's mental framework is shaped by his or her past experiences and social environment" [19] (p. 4). Researchers have found Bourdieu's work useful in understanding people's dietary habits in regard to gender [20], diabetes [21], maternal obesity [11], and food insecurity [22]. For example, Cockerham used Bourdieu's concepts of habitus to explain Russian men's unhealthy lifestyle practices: their normative behaviours of heavy drinking, high levels of smoking, high-fat diet and little or no leisure-time exercise are routine for many Russian men [23].

Most women know breastfeeding is "better" for the baby [24]. However, knowledge does not translate into action. Bourdieu shows that most of our daily lives are accomplished in a practical, unreflective fashion [16]. Bourdieu's concept of *dispositions *relate to preferences, the tendency to take up a collective way of behaving or knowing. Cultural norms are passed on to the next generation through unconscious memories of attitudes and practices [20]. Mothers strongly influence the diet of their family. The mother is usually the family food preparer and *her *intake of fruit and vegetables predicts family members' fruit/vegetable intake [25]. We have found that mothers with a higher intake of fruit and vegetables are more likely to breastfeed than mothers with a lower fruit/vegetable intake [26].

If women have grown up in communities where formula feeding is the norm, according to Bourdieu these norms of attitude and practices form unconscious memories [20]. Indeed, Groleau and Rodriguez described formula feeding in their disadvantaged French-Canadian community as the 'normal' or even 'natural' way to feeding a baby, and therefore breastfeeding as 'unnatural' or 'controversial' [12]. In this situation, many women felt disempowered by the stigmatising discourses of health professionals: "Don't you want the best for your baby?" [12]

Lee found most women (57%) in her UK study who decided to formula feed prior to the birth stated "nothing/no-one had influenced their decision" [27] (p. 474). The majority of women in Lee's study experienced "formula feeding as a pragmatically advantageous option for feeding babies" [28] (p. 12). In a recent US study, African-American women had higher 'comfort with formula feeding' which explained 37% of the disparity in breastfeeding intentions between African-American women and non-African-American women [29]. Formula feeding was normative for these women, like the behaviour of the Russian men in Cockerham's paper [23].

The promotion of breastfeeding in hospital encourages new mothers to begin breastfeeding however at home, mothers may turn to infant formula as this is how their community sees infant feeding - milk in bottles. These unconscious memories may be more influential than education and advice from hospital staff. Low income mothers were more likely to cite "*Breast milk alone did not satisfy my baby*" as important in their decision to stop breastfeeding than higher income mothers in the US [30], thereby demonstrating how they interpret the baby's behaviour through the lens of their own unconscious understandings. Women whose family and social environment is characterised by anti-breastfeeding discourses were more likely to abandon breastfeeding and adopt formula feeding to protect their physical and emotional wellbeing [12] (p.95). Whereas more educated families value optimum nutrition, antibodies, brain development and avoidance of 'second best' (ie. infant formula) [24].

Using theories, such as Bourdieu's concepts of habitus and dispositions, may aid our understanding of why breastfeeding rates remain low in many groups and why many interventions have failed. Qualitative studies are needed to explore lay beliefs about infant feeding and to test the potential usefulness of Bourdieu's theories in this area. Following these studies, researchers may be able to use appropriate theories to design intervention studies aimed at increasing breastfeeding initiation and duration in communities where formula feeding is the norm. As Coveney has stated:

"... for too long public health nutritionists have paid more attention to a universal, science-based understanding of food which they attempt to impart to clients and communities without an appreciation of lay knowledge, its social origins and the role it plays in structuring worldviews." [13] (p. 296)

Fagerli and Wandel conclude: "... it is important to be aware of factors that may act as potentials or obstacles for the consumer (or the patient when in a general practice setting) to make the necessary changes or adopt the requested behaviour in order to achieve good health" [20] (p. 188).

While a plethora of studies have been published on the determinants of breastfeeding, fewer have been published on the social meanings of infant feeding. Just as teams of researchers include a statistician to help with the design and analysis of quantitative studies, we should consider working with researchers with social theory skills to improve qualitative research in our area [31] and hence improve public health outcomes.

## Competing interests

The author declares that they have no competing interests.

## Authors' contributions

LHA wrote the paper.
